# Grant Report on mCARE: Mobile-Based Care for Children with Autism Spectrum Disorder (ASD) for Low- and Middle-Income Countries (LMICs)

**DOI:** 10.20900/jpbs.20210004

**Published:** 2021-02-26

**Authors:** Munirul M. Haque, Masud Rabbani, Dipranjan Das Dipal, Md Ishrak Islam Zarif, Anik Iqbal, Shaheen Akhter, Shahana Parveen, Mohammad Rasel, Golam Rabbani, Faruq Alam, Tanjir Rashid Soron, Syed Ishtiaque Ahmed, Sheikh Iqbal Ahamed

**Affiliations:** 1 R.B. Annis School of Engineering, University of Indianapolis, Indianapolis, IN 46227, USA; 2 Ubicomp Lab, Department of Computer Science, Marquette University, Milwaukee, WI 53233, USA; 3 Institute of Pediatric Neurodisorder & Autism (IPNA), Bangabandhu Sheikh Mujib Medical University, Dhaka 1000, Bangladesh; 4 National Institute of Mental Health, Dhaka 1207, Bangladesh; 5 Neuro-Developmental Disability Protection Trust, Dhaka 1215, Bangladesh; 6 Telepsychiatry Research and Innovation Network Ltd, Dhaka 1215, Bangladesh; 7 Department of Computer Science, University of Toronto, Toronto, ON M5S 2E4, Canada

**Keywords:** Autism Spectrum Disorder (ASD), Remote Experience Sampling Method (RESM), Value Sensitive Design (VSD), Mobile-Based Care (mCARE)

## Abstract

In low- and middle-income countries, especially in Bangladesh, Autism Spectrum Disorder (ASD) may be considered an anathema, and social-cultural-financial constraints mean that there are few facilities available for treatment for ASD children. The revolution in the use of the mobile phone (~80%) by the majority of people in Bangladesh in recent years has created an opportunity to improve the overall scenario in the treatment or remote monitoring process for children with ASD. In this grant project, we planned and developed a mobile phone-based system to remotely monitor children with ASD and help their treatment process both at the caregiver and care practitioner ends. In developing mCARE, we utilized a Remote Experience Sampling Method to design, build, deploy, and study the impact of mobile based monitoring and treatment of children with ASD in Bangladesh. We developed a mobile application using the Experience Sampling Method (ESM). A caregiver routinely reported the behavioral and milestone parameters of their children with ASD. The care practitioners monitored the longitudinal data that helped them in decision-making in a particular patient’s treatment process. The Value Sensitive Design (VSD) was used to make this mobile application more user friendly with consideration of the local economic, social, and cultural values in Bangladesh.

## INTRODUCTION

About 1% of the population in the USA and abroad [1,2], including Bangladesh [3–5] is affected by Autism Spectrum Disorder (ASD). For the larger population this number is likely to be underestimated [5,6] and is widely considered to not be less than 1%. During the last 50 years the worldwide predominance of Autism Spectrum Disorder (ASD) has expanded 20–30-fold. Bangladesh, much in the same way as other low- and middle-income countries (LMICs), is encountering an enormous gap between the care needs of families raising children with ASD and the current practitioner-family care mechanisms available to them. The objective of our work was to develop a dynamic system to improve the caregiving of ASD children in LMICs by a mobile-based application called mCARE. This system is also used for evidence-based decision making. mCARE was developed by considering three major issues in the care system: (i) financial crisis in the LMICs that limit access to children with ASD to care centers, (ii) a smaller number of care practitioners available in the care center, and (iii) limited availability of longitudinal data of behavioral changes during patient’ visits. mCARE was developed according to the socio-cultural and existing care process norms, and was used by 8 mental health professionals (MHPs) and 300 caregivers of children with ASD (CCwASD) for over one year. CCwASDs used mCARE (mCARE-APP/mCARE-SMS version) to regularly provide customized behavioral and developmental progress parameters as selected by the MHPs for their child. And on the other hand, MHPs utilized the web-based data visualization platform known as mCARE-DMP (Data Management Platform) to collect and compare the submitted data and support evidence-based decision making. The key contributions of mCARE are as follows: (i) mCARE is the first of its kind mHealth application to develop a longitudinal parametric database for children with ASD; (ii) mCARE strengthens the current care system with an evidence-based and data-driven remote behavioral monitoring platform for care practitioners, and; (iii) mCARE uses Novel Application of Experience Sampling Method for ASD through Value Sensitive Design and Fogg’s Behavior Model.

## BACKGROUND

Autism spectrum disorder (ASD) is a condition that influences communication, social interaction, behavior, and interests [7]. Information from the U.S. Centers for Disease Control and Prevention (CDC) shows the pervasiveness of ASD is roughly one of every 54 children. People diagnosed with ASD have restricted behavioral functions, such as stereotype behavior or inability to adjust to new situations [8]. Kids with ASDs often exhibit more than one core ASD symptom, and many also suffer from associated symptoms, such as severe tantrums or sleep problems [9,10]. Regardless of gaining remarkable progress in ASD screening and raising awareness in recent years, the healthcare support system is limited for families raising children with ASD in Bangladesh [11].

In Bangladesh, social-cultural-financial constraints and a scarcity of mental health care practitioners have deprived families raising children with Autism Spectrum Disorder (ASD) from regular monitoring, care, and support [12–15]. The treatment and care of kids with Autism Spectrum Disorder (ASD) are underdeveloped in Bangladesh, like other low- and middle-income countries. Existing practices depend on traditional “on the spot” evaluations of the behavior of children with ASD or caregiver recollections during their occasional and infrequent visits to a limited number of mental health care professionals. Most of the research work in this field has been done for English-speaking people or developed countries [16–20]. Research in this domain in LMICs countries has mostly been limited to screening children with ASD [21–23] and making computer games [24–27].

Caring for children with ASD is challenging and impacts family life [28,29]. The satisfaction of needs of affected children requires a lot of time, patience, and exertion, and can cause depression, anxiety, and psychological distress among their parents and caregivers [30–33]. Additionally, numerous guardians face budgetary issues, given high out-of-pocket healthcare expenses, underemployment, or employment loss [31–34].

Current treatment methodologies for ASD focus on managing conditions and improving functioning for patients as there is currently no cure [35]. There are four major treatment branches to manage ASD symptoms: behavioral approaches, dietary approaches, pharmaceutical therapy, and complementary and alternative health care approaches [36]. Behavioral approaches are frequently based on helping children figure out how to manage and communicate the way they are feeling. One broadly acknowledged conduct approach is applied behavioral analysis, in which positive and negative behaviors are encouraged and discouraged, respectively [36]. Among all the treatment strategies proposed, behavioral methods to deal with symptom management are the most common for managing ASD in children [37].

An increasing amount of research indicates strong possibilities for the use of digital interventions to support the management of the autism using computers, smartphones, wearable technologies, virtual reality, robotics and tablets [38]. Mobile solutions to a number of problems have been well accepted in Bangladesh [39] and in other LMICs [40]. Our systemic review and metanalyses prior to the project launch documented the potential effectiveness of digital health interventions in ASD.

mCARE is the first mobile-based tool to routinely and systematically collect behavior and developmental parameters for children with ASD in LMICs. mCARE is customizable to fit each child’s specific parameter monitoring requirements. This culturally appropriate application will serve an area of great need—families raising children with ASD in resource-poor countries. mCARE provides the first clinical decision support system for the care practitioners of ASD in LMICs that will assist them in data-driven decision making. Besides, this will promote a shift from the current practice of assessment from a single visit, towards an evidence-based remote monitoring and a real-time intervention paradigm. Therefore, it will expand the reach of mental health care to those who previously had little or no access. Moreover, the analysis and comparison features on the longitudinal behavior database will promote novel behavior research in the domain of ASD in LMICs. mCARE introduces a unique ESM application by applying this methodology for assessing the behavioral progress to the essential yet understudied population—children with ASD in LMICs. Unlike most apps of ESM, where the patient self-reports, we introduce caregivers as the reporter. To incorporate local norms with ESM, mCARE adopts the VSD [41] strategy to keep the existing value-system intact while improving accuracy and efficiency, and FBM [42] strategy to include behavioral persuasion to encourage caregivers to use mCARE regularly. Thus, mCARE builds on a set of established concepts and methods with novel applications in scarce resource contexts.

## KEY CONTRIBUTIONS

The main contributions of mCARE are:

- **Provides a novel mHealth system for children with ASD that includes a longitudinal data visualization application:** Some studies have been done, and several applications were developed for children with ASD [43–45]. mCARE is the first mobile-based tool to routinely and systematically collect behavior and developmental parameters for children with ASD in LMICs. For monitoring the mental health condition of children with ASD, a set of specific and routinely monitoring parameters are required for every child. Here mCARE provides such customizable options both for the caregiver and care practitioners. In this system, they can set and give the necessary parameters for each patient based on their mental condition and goals for development. The application mCARE:DMP plays a vital role in generating web-based data visualization and comparison of the real development of an ASD child from the longitudinal parametric data. This culturally appropriate application can serve in areas that do not have adequate resources and facilities where families face difficulties raising children with ASD.
- **Qualitative and quantitative impact of data and feasibility study:** One primary condition for the development of children with ASD is the practical use of longitudinal monitoring of the symptoms continuously and regular feedback from care practitioners. Existing “on the spot” assessments of children with ASD symptoms may pose difficulties because of irregular and infrequent visits to care practitioners. Many parents cannot provide their children with high levels of support, Practitioners themselves are often not well trained in how to deal with the massive amount of data when it is available [46]. In this regard, mCARE plays a vital role in providing qualitative and quantitative data at regular intervals, reducing the complexity of its potential use. mCARE was designed so that care practitioners can use this qualitative and quantitative data promptly and adequately for evidence-based decision making.
- **Design challenges and implications for effective behavioral data collection:** Our primary focus behind developing the mCARE system was to help doctors for treating children with ASD in LMICs, especially in Bangladesh. mCARE was designed and developed based on the data collection environment of Bangladesh. At the end of March 2019, the number of mobile phone subscribers reached 159.780 million in Bangladesh [47]. As Bangladesh is a developing country, many people do not have access to smartphones; instead, they use feature phones. For our study’s data collection, we developed an app for the smartphone user group and an SMS system for those using feature phones. 60% of app users and 40% of SMS users participated in our study. Our data collection phase started in November 2019 and ended in November 2020.

## RELATED WORK ON MENTAL HEALTH USING MOBILE TECHNOLOGIES

Prottoy [48] is a smartphone-based application to identify autism in children at the early ages for low- and middle-income countries like Bangladesh. This mobile-based application was designed for the Bangladeshi people in the Bengali language. This interactive automated and 3D scenario-based application used Childhood Autism Spectrum Test (CAST), and Cross-Cultural Translation & Validation model, and employed 39 questions and some 3D characters and scenarios for detecting autism in children aged 3–11 years in Bangladesh. This application also recommended the nearest ARC/hospital treating children with autism. A main problem of this work was in detecting false-positive results of Autism children.

In [49], Kim et al. summarize and categorize all smartphone applications for Autism Spectrum Disorder (ASD) from the Autism Speaks official website [50]. On that website, ASD related apps are assigned research ratings in three categories: (i) Anecdotal: with this rating no specific studies are associated with this type of app, (ii) Research: in this category, some targeted studies were found but no direct research studies were found and, (iii) Evidence: Under this category, apps are based on solid and specific research evidence. Kim et al. found 695 apps on ASD, where only 40 apps were based on solid and specific research evidence.

Bangerter et al. created a web-based mobile health (mHealth) app known as My JAKE (Janssen Autism Knowledge Engine), which helped caregivers continuously monitor the treatment of autism-related symptoms and track the progress of their children [51]. A total of 144 patients were recruited for this study. Recruited patients’ age range was six to adult. Caregivers of individuals with ASD used the My JAKE app to make daily reports on their child’s activity and other self-selected specific behaviors. The observational period of this study was 8 to 10 weeks. The results were analyzed with paper-and-pencil scales obtained over a simultaneous period at regular 4-week intervals. My JAKE successfully captured the caregiver reporting of behaviors in real-time. Caregivers sent feedback 2–3 days per week across the study period. But some limitations were also seen in their system. In the system, there was no way to change the behavioral parameter questions dynamically. For treatment purposes, care practitioners were not able to change some parameters. Moreover, there was no feedback system or sending a periodic report to the caregiver.

Baio et al. discuss a network system known as Autism and Developmental Disabilities Monitoring (ADDM), an active surveillance system [52]. This system estimated the prevalence of autism spectrum disorder (ASD) among children aged 8-10 years. This study was done at 11 Autism and Developmental Disabilities Monitoring (ADDM) sites in the United States. The work was carried in two phases. In the first phase, professional service providers in the community did the review process and abstraction of comprehensive evaluations. For study participants who received special education services in public schools, most of the ADDM sites also reviewed previous records for them. To determine ASD case status, all information was systematically examined by experienced clinicians. This work was done in the second phase of the project. If a child displayed aberrant behaviors and if he/she met the surveillance case definition for ASD, then one or more comprehensive evaluations were completed by community-based professionals. This work was based on the Diagnostic and Statistical Manual of Mental Disorders, Fourth Edition, Text Revision (DSM-IV-TR) criteria. It presented updated ASD prevalence assessments for children aged eight years during the 2014 surveillance year, representing characteristics of the population of children with ASD.

Akhter et al. proposed a cross-sectional study in [53], where they tried to find out the early detection of Autism Spectrum Disorder (ASD) symptoms among children aged 18–36 months. This study explored a rural community of Bangladesh and was conducted among 5286 children in that rural area. The main methodology was to collect the household level data with the screening tool MCHAT, and by this tool primarily selected 66 children positive for ASD. After that, they invited those 66 children for final diagnosis, where screening was made by both MCHAT and flashcard, and by the pediatric neurologists using diagnostic tools (DSM-IV & ADOS). And among the 66 children, four children were identified with ASD. By this study, the authors claimed that in the rural area in Bangladesh, the ratio of the prevalence of ASD is 0.75/1000. They also asserted that early detection of ASD in rural communities could help to decentralize decision making in health services among children with ASD.

Khan et al. developed an autism spectrum disorder (ASD) intervention application named MyHeifer [36]. Their work aimed to recognize patients’ behavioral patterns properly, make healthcare decisions, ease caregiver burden, and provide an emotional outlet for patients. The MyHeifer application helped children with ASD to express and explore their emotions within an uncomplicated environment. Children performed some actions or interactions through the app, which were classified as either positive or negative behaviors. The choices children made were collected and served as a basis for future healthcare decisions. As it is challenging to communicate with children with ASD, using data from past actions or interactions helped caregivers understand the challenges of making better emotional and behavioral connections with the children. This application mainly served as a medium for addressing emotions and behavioral decisions.

In [54], Sharmin et al. proposed a set of implications guidelines for designing technology-based Autism Spectrum Disorder (ASD) intervention by analyzing 149 peer-reviewed articles (119 works from developed countries and 24 works from developing countries) related to ASD from a digital library. They found different levels of technologies: (i) wearable devices like a smartwatch, wrist/chest/ankle bands, head-mounted displays, and cameras (51 papers), (ii) Smartphone, tablet, iPad or mobile applications (58 papers), (iii) Virtual Reality (VR) (11 papers), (iv) Robots (14 papers). For making these kinds of smart innovations for the ASD patient, the most common features were: culture-driven design of the technology, selecting the essential physiological signal like ECG, GSR, etc., capturing the history of the treatment plan, and designing the appropriate feedback mechanism. Though therapy is the most effective way to improve ASD conditions, technology also plays an important role by collecting real-time interaction data to create a rich history where the most important changes of an ASD patient can be stored. These captured histories help the therapists to take evidence-based decision making in the treatment process.

The work presented in [29] was about Autism Spectrum Disorder (ASD) in low- and middle-income countries (LMICs), where unique challenges were presented as cultural misperceptions, and social practices often impeded effective care. The mobile phone adoption in many LMICs has grown over time; it has created a suitable opportunity for enhancing ASD care practices through digital means. Qualitative findings on the challenges of designing mobile assistive technologies for ASD in Bangladesh were presented in this study. Technical, social, and cultural difficulties were discussed to develop a system for caregivers and care professionals. Some design aspects were also discussed to overcome the challenges.

## METHODS

We followed a multi-phase mixed method study design, summarized in Table 1. This table describes the timeline (2 years or 24 months) of mCARE’s phases including the method names and participant numbers (here C for Caregiver and P for Practitioner). All the steps of mCARE were developed in a certain time frame and the same grey scale means the same participants were used. Here the columns represent the different phases of this study and rows represent different milestones with the method name.

**Figure 1.**
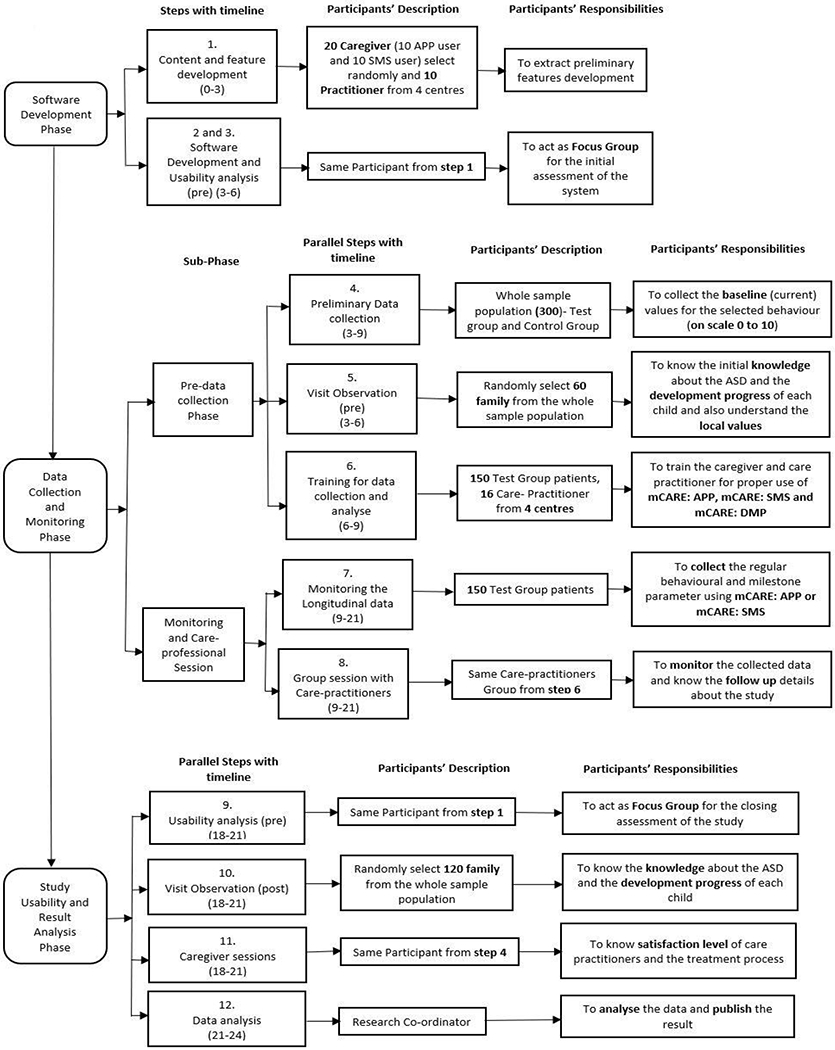
A screenshot of mCARE flow chart diagram with different phase with participates description.

The overall flow diagram of mCARE with the participant’s description from Table 1 is shown in Figure 1. We can divide our study into three phases: (i) Software Development, (ii) Data collection and Monitoring, and (iii) Study Usability and Result Analysis. In our whole study, there are mainly two types of participants: (i) Caregivers (the parents of the children) and (ii) Care practitioners (the clinical coordinators of the centers). At the end of the study, the research coordinators were responsible for analyzing and publishing the significant result and findings. In our study, some parallel tasks (in phase ii and iii) were done based on the demand for this project’s progress in the project timeline (shown in Table 1). We formed a different group of participants for different steps from our sample population based on our project’s demand and accomplishment. Each group had a particular role in each step (details are shown in Figure 1). In the first phase of software development, the main goal was to extract all features for the development, complete the whole system as ready to use, and analyze the system’s initial usability. We complete this phase by three different steps with the help of twenty caregivers (10 APP and 10 SMS users), who were selected randomly, and ten care practitioners (the clinical coordinators) from the four centers. The second phase has a total of five steps into two sub-phases. In the pre-data collection phase, we collected the participant’s knowledge of ASD, their baseline data and trained them (especially the test group caregiver) to use mCARE tools (mCARE: APP and mCARE: SMS). On the other hand, in the monitoring and care practitioner session, we collected the test group’s longitudinal data, monitored and followed by the care practitioner. In the last phase of this study, we determined the satisfaction level, the progress of the ASD patient, knowledge of ASD; and overall data monitoring, analyzing, and publishing the findings.

This study took place at 4 major institutes of Bangladesh in two geographical locations—Dhaka and Chittagong. We tested the mCARE system at two government organizations: National Institute of Mental Health (NIMH) and Institute of Pediatric Neurodisorder and Autism (IPNA). The participants were divided into two groups—mCARE-APP (50) and mCARE-SMS (50). Moreover, each group was divided equally in test (25) and control (25) groups. Typically, in Bangladesh, families with low and high socioeconomic resources receive treatment from public and private organizations respectively. To include participants from all socioeconomic classes, we included two private organizations—Nishpap and AWF, and 50 participants were recruited from each of these schools. In the study, the control group patients were selected randomly from the total population of the study and were not monitored regularly through mCARE like test group patients. In this study, after every three months the clinical coordinator contacted control group patients for their feedback about the milestone and behavioral parameters. This data was used to compare the differences between the test group and control group.

To assist in the mental development process, we proposed a novel method to help both caregiver and care practitioners continuously and regularly monitor the ASD children. Broadly, mCARE has two main phases: Data Collection and Data Analysis.

### Phase 1: Data Collection Phase

The initial phase of mCARE is data collection. For data collection we implemented mCARE at 4 major institutes of Bangladesh in two geographical locations—Dhaka and Chittagong. We collaborated with two government organizations for ASD treatment and research, NIMH (National Institute of Mental Health) and IPNA (Institute for Pediatric Neurodisorder and Autism), to recruit 100 caregivers of children with ASD from each. The participants were divided into two groups—mCARE-APP (50) and mCARE-SMS (50). Each group was further divided equally in the test (25) and control (25) groups. Typically, in Bangladesh, families with low and high socioeconomic resources receive treatment from public and private organizations, respectively. To include participants from all socioeconomic classes, mCARE also included two private organizations—Nishpap Autism Foundation and AWF (Autism Welfare Foundation). Fifty participants were chosen from each of these schools, divided into test (25) and control groups (25) only for the mCARE-APP study. A summary of the participants is given in Table 2.

A total of 300 children, aged 2 to 9, were involved in this project. We incorporated diversity in terms of age, sex, ASD severity, and family socioeconomic resources. The power analysis yields that the sample size of 300 is sufficient to discover a difference among mCARE-SMS, mCARE-APP, and the control groups with the power of 0.97 at α = 0.05 and the medium effect size.

## DEVELOPMENT OF mCARE

Software development for mCARE-SMS/mCARE-APP occurred in three cycles over three months. For each period, the first step was analyzing the study requirement. Next, the software program was designed with the research team’s input and vision. Determination of the sequencing of events in the system and the flow of information were made. Later, the software program was coded. A minimal viable product of the software system was built and tested to identify flaws. After collecting feedback from ten caregivers, system adjustments were made, and the process of analysis, design, coding, and building a prototype was repeated. Further, we evaluated options for providing written instructions, audio guidance in the local dialect, or video in which a mother of a child with ASD showed the steps [55,56].

We conducted a formal heuristic evaluation and cognitive walkthrough of mCARE using Nielsen’s heuristic evaluation to ensure the usability and navigability of the developed tool [40]. Once we had a final design, we conducted standard usability tests to measure the time taken and the number of errors made for one complete submission of data using mCARE-SMS/mCARE-APP. We also used the System Usability Scale (SUS) to validate the user interface design with questions on the usability, learnability, complexity, navigability, and confidence of use [57]. Ten caregivers (different from those who gave feedback during the evolution phase) were randomly used for this usability study of mCARE-SMS and mCARE-APP. We also considered different design trade-offs, such as a login-based method (more secure but less user-friendly) vs an IMEI-based authentication method (less reliable but provides ease of use).

One of the challenges mentioned by the caregivers is that they did not have an idea of what to achieve next after a certain point. As a solution, we added developmental progress parameters as part of the longitudinal monitoring to be used as individualized milestones. Therefore, caregivers could update the profile with new milestone goals to be achieved, thus engaging them with small targets. The answers to the questions of these parameters were recorded as ‘yes/no’ monthly. We sent a bi-weekly progress report to the caregivers whose content was finalized based on caregiver and care practitioner input. Overall, it summarized the child’s performance in the last month and outlined any odd value if represented. It allowed caregivers to reflect on the condition of their child. Practitioners could add/delete new parameters to be monitored, and they also could change the monitoring frequency based on the situation.

We recorded the proper consent from the caregivers with their information in the data collection phase. On the consent form, we included information on what data we would be collecting from them over the one-year project span of mCARE. We collected two types of data (*Behavioral data and Milestone parameters*) from the caregivers from both the test and control groups. The main difference between these two groups is that we collected regular data from the test group, where we only collected data from the control group once every three months.

- Behavioral data for ASD patients in mCARE: Individual behavioral parameters were collected from the test group regularly, for example: Repetitive activity (such as spinning a pencil/ hand flapping), inflexible to change, repetition of same word, use of meaningless word, use of pronouns inappropriately (such as “I” instead of “you”), use of unnatural sounds (e.g., high pitch squeal), can s/he start social interactions, etc.
- Milestone parameter for the ASP patients in mCARE: Like the behavioral parameters, we also collected milestones parameters in four clusters: *Type 01: Communication:* (points to at least 5 body parts when asked, listens to a story for at least 15 minutes, follows instructions in if-then form (e.g.,: if you want to play outside then finish your food first), says 1st and last name when asked, etc.), *Type 02: Daily Living Skills:* (urinates in toilet/potty, asks to use toilet, can zip zippers that are fastened at the bottom (e.g., pants or backpacks), puts shoes on correct feet, etc.), *Type 03: Socialization domain:* (answers when familiar adults make small talk (e.g., if asked ‘how are you?’ says ‘fine’), use words to express emotions (e.g., ‘I am happy’, ‘I am scared’), shares toys or possessions, takes turns when asked while playing games or sports, etc.), and *Type 04: Motor skills domain:* (runs smoothly without falling, jumps with both feet off floor, throws ball of any size in specific direction, walks up/down stairs, etc.).

### Phase 2: Data Analysis Phase

mCARE: DMP was employed for the overall Data Analysis Phase. Using the result generated by the mCARE: DMP, two levels of data analysis were completed in mCARE, which are as follows:

#### Level 1: Data Analysis by Clinical Coordinator:

**Figure 2.**
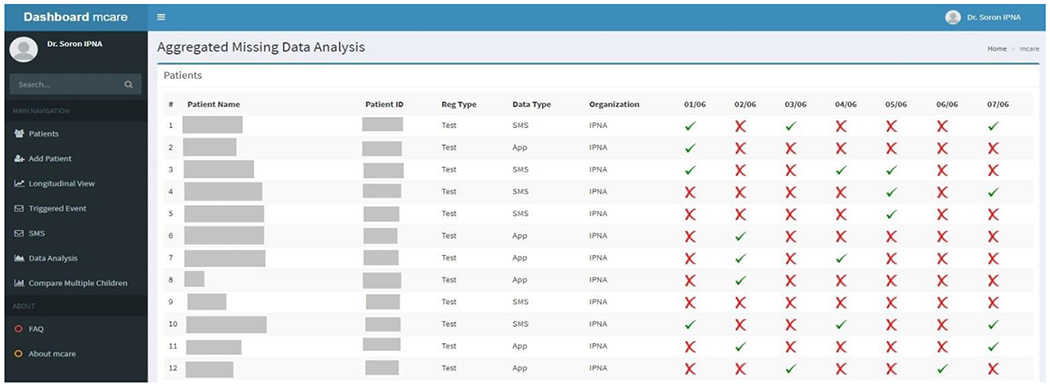
A screenshot of mCARE: DMP view for data collection.

In this step, all four clinical coordinators (care practitioners) used the data from their respective centers. A screenshot of mCARE: DMP view for IPNA is shown in Figure 2. The clinical-coordinators used these data for several purposes, including:

- For regular feedback to the patients: Every week for each center, a clinical coordinator checked every patient’s data from mCARE: DMP. The clinical coordinator regularly checked the behavioral and milestone parameter data of the patients in their center. They made evidence-based decision from the data; for example, if any data for any parameter remained satisfactory for a long time, the clinical coordinator could change the parameters and set new parameters for that patient. If the progress for any parameter remained the same or degraded, then the clinical coordinator gave some feedback for the development of that parameter. This evidence-based decision-making feature of mCARE developed more milestone parameters of the test group patients in comparison to the control group patients.
- For focus group discussion: The clinical coordinators also used the mCARE: DMP for conducting focus group discussions. For example, during the COVID-19 pandemic, a specific focus group discussion was held with the group targeted based on data analysis from the mCARE: DMP. The clinical coordinators selected their focus group from their centers and set a specific question for participants through mCARE: DMP. The category of the questions in this focus group included:
  - About usability of mCARE
  - mCARE in COVID situation
  - Motivation on mCARE
  - About Stigma
  - Satisfaction Level and Basic ASD Questionnaire in mCARE

#### Level 2: Data Analysis in the weekly meeting:

From the start of this work, we continuously and regularly met on an online platform, ZOOM [58], once per week. Weekly meetings included all four clinical coordinators, our research group members, and the co-principal and principal investigator (PI and CO-PI). The main purpose of this meeting was to make a bridge between the clinical coordinator and the researchers. These meetings achieved the following:

- Identify important findings from the collected data
- Make decisions after analyzing data and evaluate the feature accordingly
- Continuous follow up report of the patients’ data
- Show the weekly report
- Make decisions to complete the aims of mCARE

**Figure 3.**
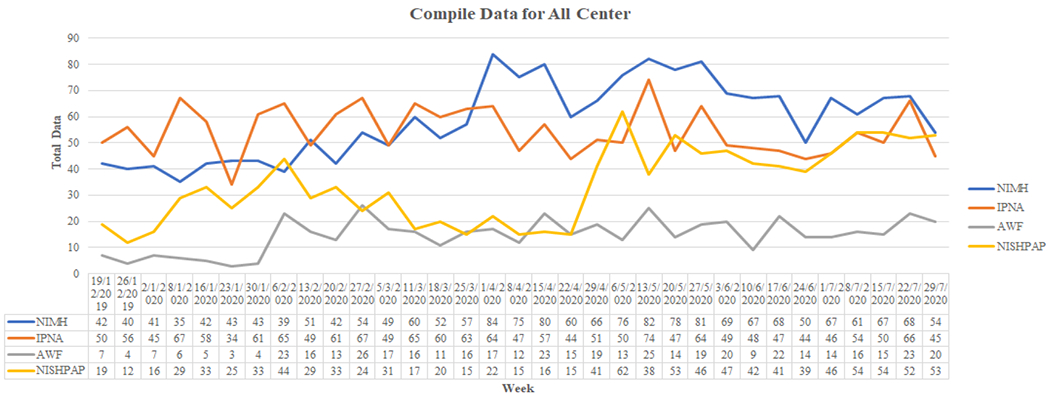
A screenshot of weekly comparison line graph for all four centers (from 19-12-2019 to 29-07-2020).

During each meeting, the team looked at all data collected from the patients during that week. Graphical representations of the data were generated by mCARE: DMP. Figure 3 shows the weekly comparison line graph for all four centers (from 19-12-2019 to 29-07-2020). This data visualization provided easily understood data rationality among the four centers. We could easily discover data variants for a particular week for any specific center.

**Figure 4.**
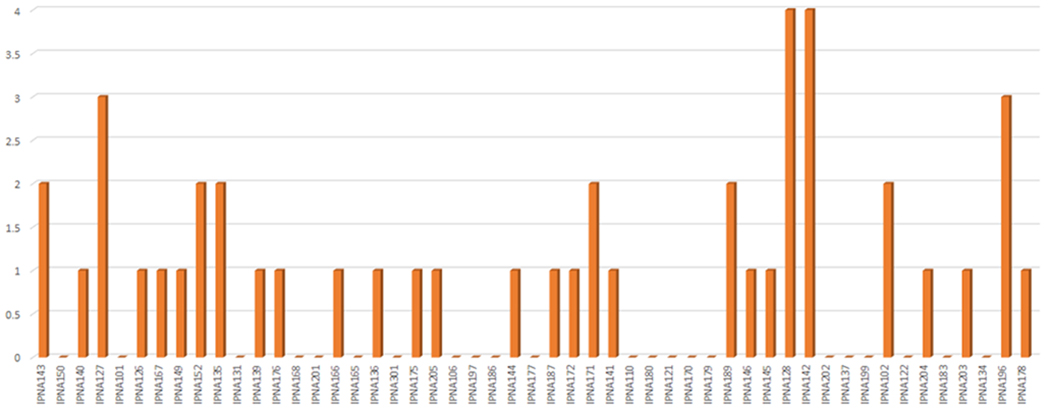
A screenshot of the center wise (IPNA) data representation generated by mCARE: DMP for every week.

Through these weekly meetings, and with visualized data, mCARE ensured regular feedback from caregivers and assisted in providing appropriate mental development plans for each of its recruited patients. Figure 4 shows the center wise (IPNA) data representation generated by mCARE: DMP for every week. This figure shows the cumulative data of every patient for a particular week of IPNA.

### Phase 3: Result

The overall outcome of this work is to create a historical dataset for children with ASD, which helps care practitioners develop treatment plans using evidence-based decision making.

## EVOLUTION OF mCARE

**Figure 5.**
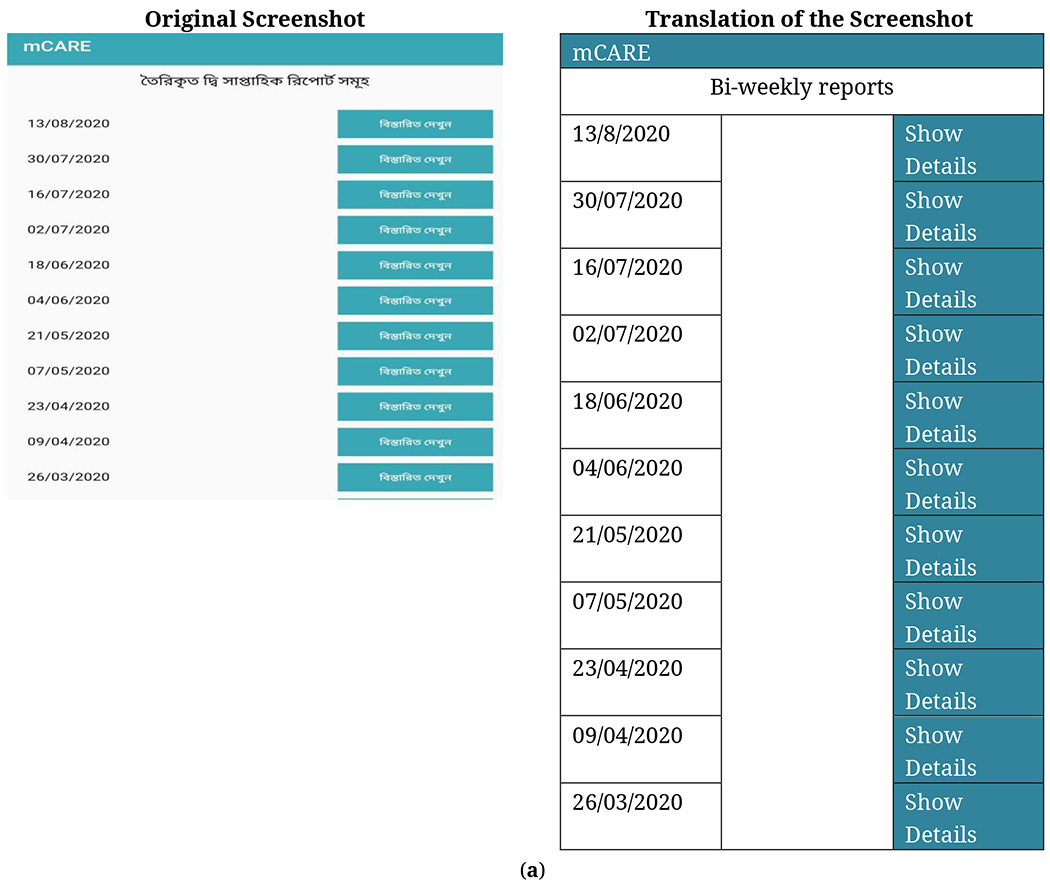

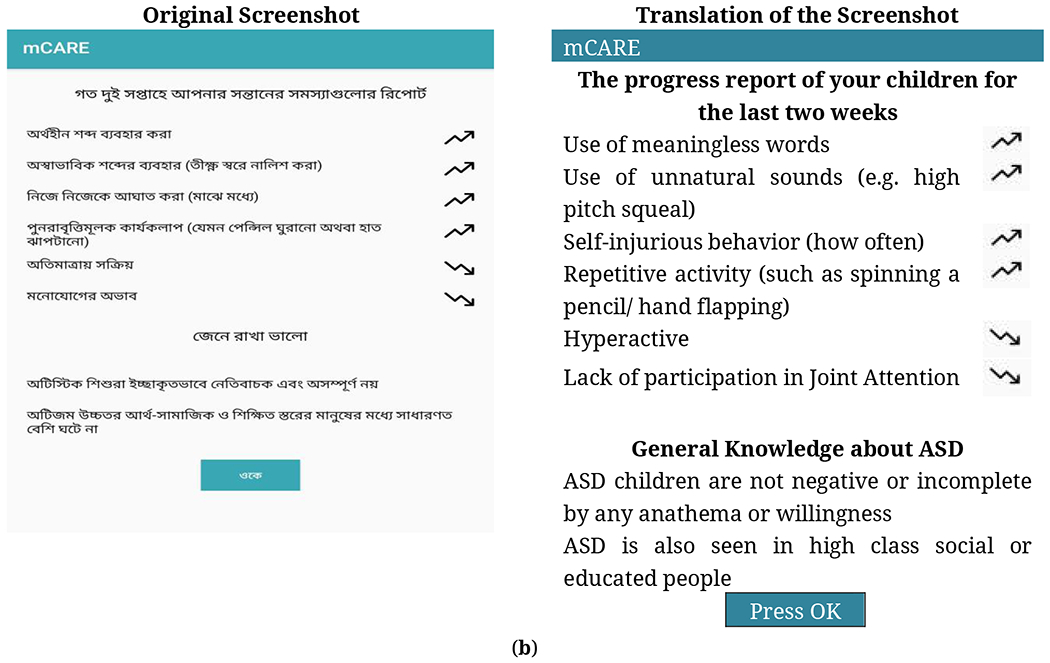
Screenshot of the progress report for a patient with translation. (**a**) Screenshot of summary patients’ feedback in mCARE:APP. (**b**) Patients’ improvement report in mCARE:APP.

mCARE was developed based on our pre-planned specifications. After starting the data collection phase, we encountered some issues and made adjustments to update our system. Some significant evolutions included:

- *Bi-weekly report generation:* In the initial stages of the development of mCARE, there was no option to provide the patient’s progress report to their parents from the system. After starting the system, for maintaining parent motivation, we developed a report generating option to mCARE, where every parent was provided a progress report every two weeks. This option helped both the caregiver and the practitioner. Figure 5a,b shows a screenshot of a progress report for a patient.
- *Update the SMS pattern:* For SMS users of mCARE, initially there was a format, comma (,) separate input, for taking the parents’ feedback to the system. After starting the data collection phase, we observed that the specific format was not always followed. For that reason, we updated the SMS pattern for the SMS based user where they can send data using space separated, semicolon separated, dash separated or mixed format.
- *Set multiple mobile numbers for one patient:* Initially, every patient was registered with one mobile number and mCARE: SMS-based users received and sent their data and feedback from that particular mobile number. After analyzing the data through mCARE: DMP, we found some missing data for a specific patient with ID: IPNA186 of the IPNA center. Our data analyst then informed the corresponding center coordinator. After an investigation by the center coordinator, we found that particular patient was registered with his father’s phone number. For some reason, his father was going away from his family, and that’s why his mother did not get an SMS from the system and also didn’t send the feedback. From this case, we realized this might happen in the future for other patients. Our development team updated the system so that a parent can add a secondary mobile number to get and send feedback if they need it.
- *Update the permission of the profile setting:* At first, only the researcher could edit any patient’s related data in the database. But parents of the patient always contacted the clinical coordinator and discussed their problems, including the difficulty of sending data. To ease the process, clinical coordinators from their respective centers were permitted to edit patient data, add alternate phone numbers, edit behavioral and milestone parameters and their frequency, and reset the password for the app.
- *Update the Behavioral parameters based on the scoring:* The clinical coordinator can change the behavioral parameter based on their treatment purpose. For instance, a clinical coordinator from IPNA noticed that a patient with ID: IPNA101 in the system had achieved a full score in a behavioral parameter. The score remained the same for a while. So, the clinical coordinator updated the parameter relevant to that patient’s treatment process.

## DATA ANALYSIS AND FINDINGS

### Demographic of the Population

In our study, we had two groups: 150 ASD children in the test group, and 150 ASD children in the control group. A summary of the demographics of these two groups is shown in Table 3.

## DATA SUBMISSION RATE

In this study, we mainly took two types of data (behavioral parameter and milestone data) from both groups. In the test group, the behavioral data were collected daily by mCARE: APP or mCARE: SMS. The weekly submission rate of behavioral data for the test group was approximately 60-70%. We took the milestone parameters from the test group once per month and the data collecting rate was about 80–90%. On the other hand, for the control group we collected the milestone and behavioral parameter quarterly, and the data submission rate for that group was about 70–80%.

## COMMON BEHAVIORAL DATA

The common behavioral parameter list is:

- Repetitive activity (such as spinning a pencil/ hand flapping)
- Inflexible to change
- Repetition of same word
- Use of meaningless word
- Use of pronouns inappropriately (such as “I” instead of “you”)
- Use of unnatural sounds (e.g., high pitch squeal)
- Can s/he start social interactions?

## EXAMPLES OF BEHAVIORAL CHANGES OVER THE STUDY

**Figure 6.**
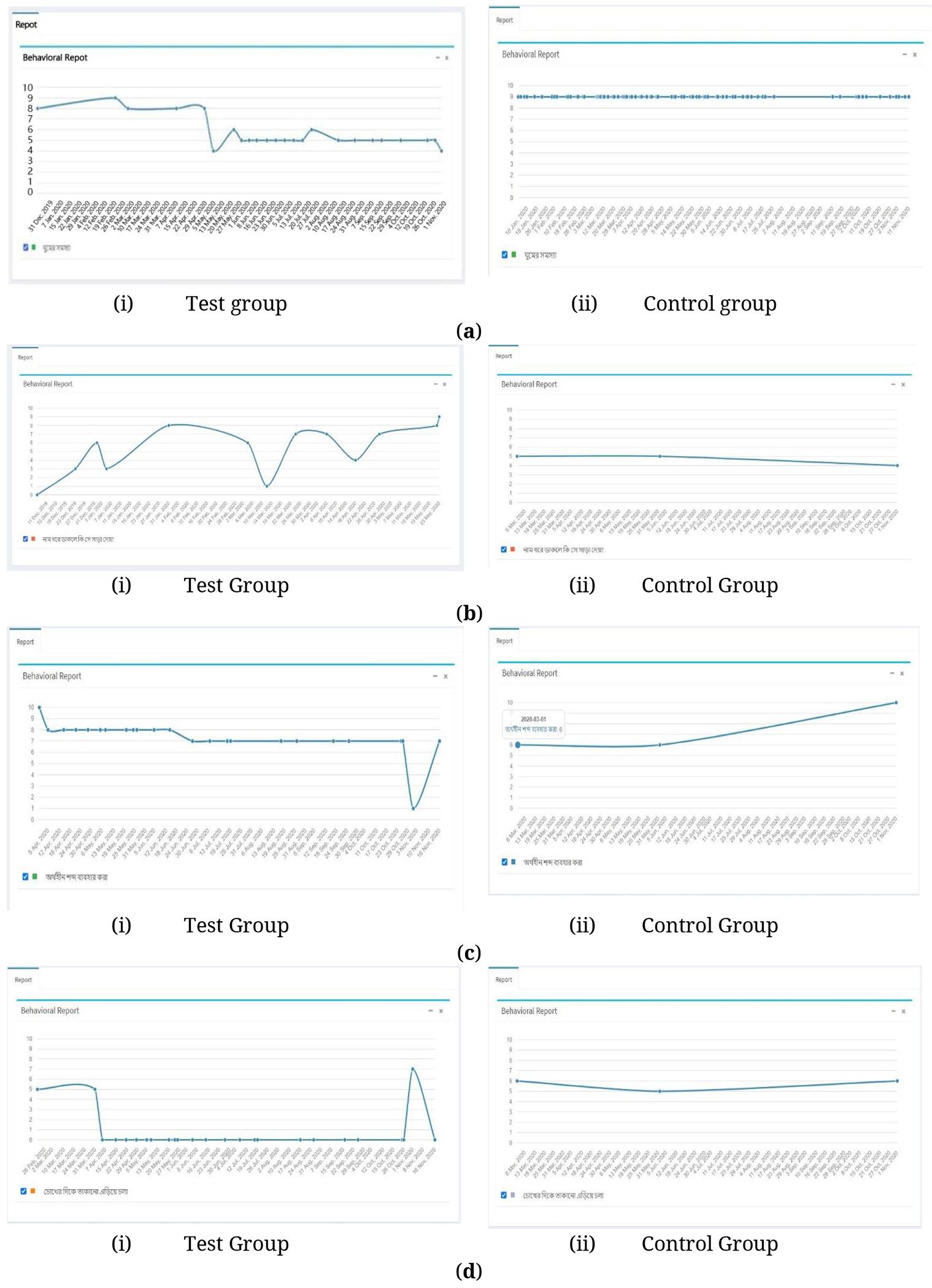
An example of the behavioral changes by mCARE. (**a**) Behavioral parameter of “Sleep Problem”, (**b**) Behavioral parameter of “Does s/he respond when called by name”. (**c**) Behavioral parameter of “Use of meaningless words”. (**d**) Behavioral parameter of “Avoid looking at”.

We observed many significant changes in study participants over the period of mCARE. In this study, we considered 32 different behavioral parameters for both test group and control group patients, but we selected 4–5 different parameters based on the condition of the patients by the clinical coordinator at the starting of the study. Here we represent four behavioral parameters from both groups which were selected randomly. Figure 6a shows a significant change in the “Sleep Problem” category, a negative impact behavioral parameter. At the start of the study, the impact of sleep problems for the test group was high; at the end of the study, the impact was significantly reduced, but for the control group this parameter was constant. Similarly, Figure 6b shows a positive impact behavioral change, “Does s/he respond when called by name?” In this case, we observed that there was a low response at the start of the study for that test group patient, but over the course of the project, the response was gradually increased, which was a positive impact for that child. On the other hand, in the control group the parameter was declined. In Figure 6c,d, we represent and compare the “Use of meaningless words” and “Avoid looking at” behavioral parameter between a test group and a control group patient.

## IMPACT OF MILSTONE PARAMETERS AND AWARENESS

The clinical coordinators observed through improvements in milestone parameters that increased knowledge and awareness of ASD by caregivers improved their motivation. We took a survey at beginning of our study among the test group parents about their ASD knowledge level. Most of them had very little knowledge about ASD. Through our focus group meetings and discussions, we educated caregivers in the study about ASD and how to manage it.

**Figure 7.**
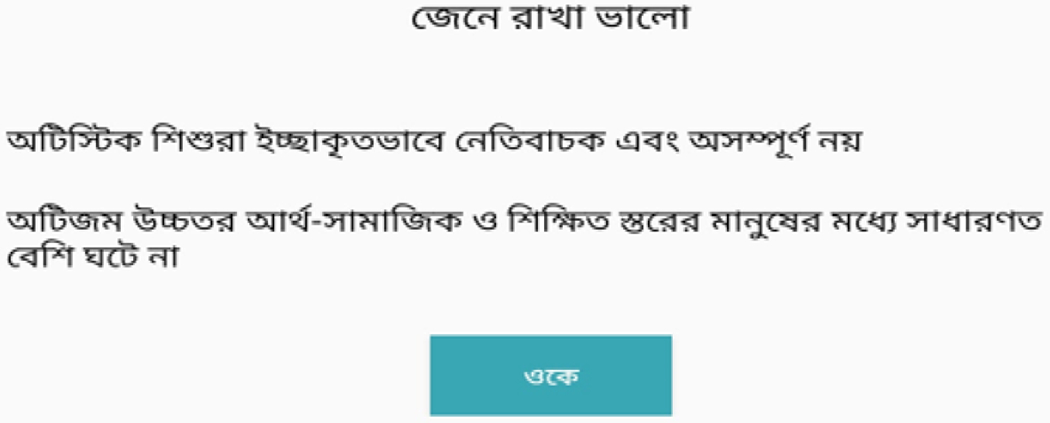
A biweekly screenshot of the tips about ASD knowledge to the App user parents.

In addition to education within focus groups, we sent biweekly feedback through the app to the user with tips (in Bengali Language) to enhance their ASD knowledge. At the end of the study, the clinical coordinators remarked on the observed improvement of caregiver knowledge about ASD. Figure 7 shows a screenshot of a tip where the message was “It is good to know that: ASD children are not negative or incomplete by any anathema or willingness. And ASD is also seen in high class social or educated people”.

## ACTION (MILESTONE PARAMETER) WITH DEMOGRAPHIC INFORMATION

**Figure 8.**
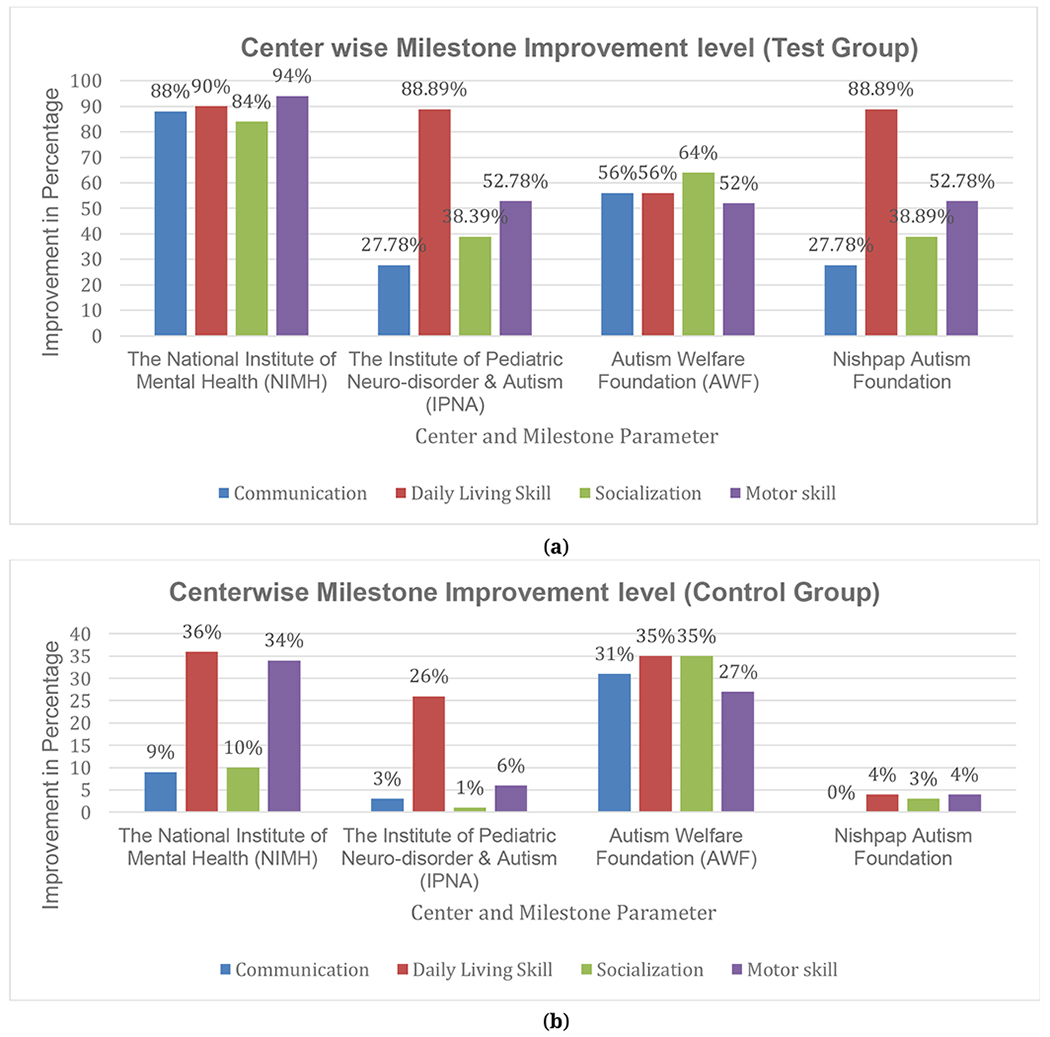
Center wise Milestone parameter improvement level for: (**a**) test group, (**b**) control group.

Clinical coordinators explored the center-wise milestone improvement of test group and control group for participants. The percentage of the improvement level from each center is shown in Figure 8a for test group patients and Figure 8b for the control group patients. In all centers, the improvement level in Daily Living skills was higher than other milestone parameters.

For Figure 8a,b, we have 150 patients (test group) and 150 control group respectively to determine the improvement level in four milestone parameters for all centers. The below Table 4 shows the 95% Confidence Interval (CI) for these two group in milestone parameters improvement level.

**Figure 9.**
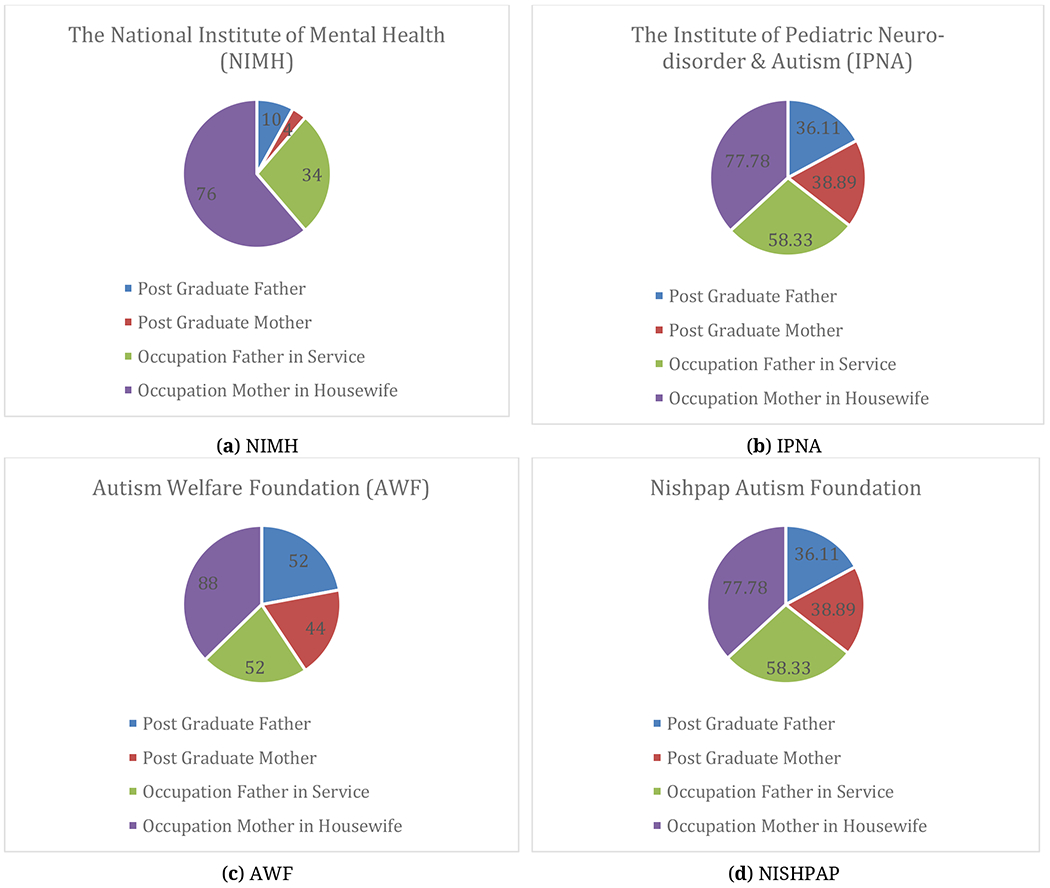
Demographic representation of the parents (Occupation and Education) for the improved test group patients.

Clinical coordinators also observed that milestone parameters were improved for the patients where families had higher levels of education rather than overall. Figure 9 shows that parents with higher levels of education, where fathers did service and mothers were housewives, their children showed more improvement than others.

**Figure 10.**
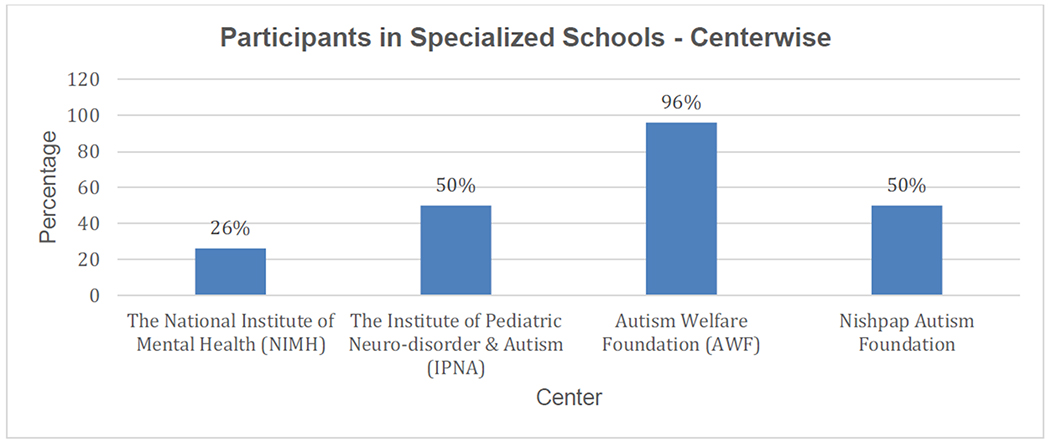
Representation of test group patient who are going specialized school for their development.

Another important finding from the milestone parameter is that the participants who attended a specialized school showed greater improvement than others. In our four centers, three had 50% or more participants going to specialized schools (shown in Figure 10).

**Figure 11.**
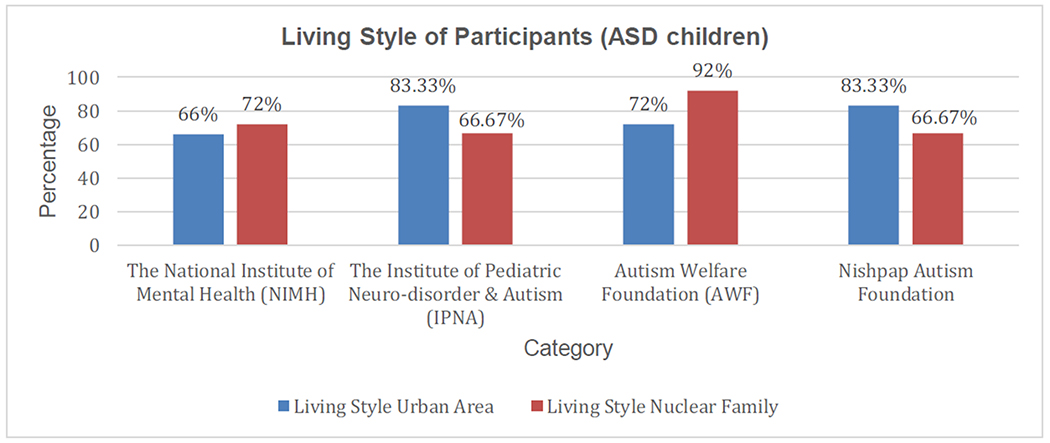
Demographic representation of living place and style for the improved test group patients.

According to our clinical coordinator, participants living in rural areas with small families (with one or no sibling) showed more improvement than other participants. Figure 11 shows the reflection of this case study. According to the clinical coordinators, the better care facilities in rural areas and opportunity to get more time from the parents in a nuclear family creates a better environment for the improvement of ASD children.

From Figures 9–11, we have shown the demographic information (who have developed their milestone parameter in test group) of parents, ASD children specialized education and their living place. By this information, we can get the idea how an ASD child be improved in an environment and which factors sounding him/her will impact to his/her mental development. Table 5 shows the Confidence Interval (CI) (for 95%) for this information.

## DISCUSSION

We have calculated the 95% Confidence Interval (CI) for the validation of our results. As our sample size was 150, we have used the “*Z*” value (1.96 for 95% CI) [59] for calculating the 95% CI using the following formula:

$$\bar{X}\pm Z\frac{s}{\sqrt{n}}$$

Where:

- $\bar{X}$ is the mean;
- *Z* is 1.96, chosen from the *Z*-value table [59];
- *S* is the standard deviation;
- *n* is the sample number.

From the Tables 4 and 5, since we have 150 participants, we can improve the result statistically significant by increasing participant number in future. Now, from the present data and result from Table 4 we can see that, the test group participants have slightly better improvement level in statistically than the control group participant. For example, in “Daily Living Skill” the 95% CI for test group is from 26.1 to 30.4 and for control group it is from 22.9 to 27.6.

Additionally, the caregivers that took part in the mCARE study were highly satisfied with its performance. According to their final feedback, the continuous and routine monitoring of their children through mCARE-APP or SMS increased their motivation. Furthermore, due to the pandemic of COVID-19, the mCARE system was perfect for monitoring participants through remote methods. The system performed well with the social and economic constraints that the pandemic placed on participants. We found a number of impacts directly related to COVID-19 on the participants and caregivers through the mCARE system. Without mCARE, we would not have that data. There were some difficulties related to data collection due to the pandemic. During the lockdown period, we observed that many parents lost their jobs and business, directly affecting family income. Some parents shifted their homes from towns to villages. Others were fully locked down at their home. For these reasons, the caregivers could not always manage to submit data, and we had to manually remind them to submit the data regularly. During the long lockdown, the participants could not go outside their home, go to school, or be seen in clinics, resulting in some instances of anxiety. We can see these results in our data. Though we have finalized our project, the data we have acquired through it will be a great asset for future research on the area and in the development of ASD children, especially in LMICs.

## STUDY LIMITATIONS AND FUTURE DIRECTIONS

Though we have some great and innovative findings from this study, we faced some challenges throughout. First of all, caregivers wanted to see immediate development and improvement of their child through mCARE. When some parents did not see improvement immediately, they were sometimes demotivated. As a result, sometimes they did not provide their child behavioral data regularly. Therefore, it was difficult for the care practitioners to make evidence-based decisions by using this system. Another challenge was the lack of proper training sessions with the parents due to the lockdown period of COVID-19. We hope to resolve these issues in our next extension (in R01) of this project.

## CONCLUSIONS

The mobile-based care using remote experience sampling method (mCARE) in the ASD children monitoring and treatment process is suitable, and it can be used in both developed and LMICs. During the study period, we found some great findings in both behavioral and milestone parameters of ASD children. We have a greater number of milestone parameter improvements in our test group patients than the control group. Both the caregiver and care practitioner were highly satisfied to use this system in the treatment process of ASD children. We believe mCARE can play a significant role in the improved development of children with ASD, as well as reveal current unknowns about this condition.

## STUDY TIMELINES AND MILESTONE

The study timeline and milestones for mCARE is shown in Table 6. This study took place over a 2-year time duration.

## Figures and Tables

**Table 1.** Summary of study design.

mCARE	0–3	3–6	6–9	9–18	18–21	21–24
**Content and feature development** Method: Interviews, focus groups	*n* = 20 (C) *n* = 10 (P)					
**Software development** Method: VSD, FBM		*n* = 20 (C) *n* = 10 (P)				
**Usability analysis** Method: Nielsen’s heuristics, System Usability Scale		*n* = 20 (C) *n* = 10 (P)				
**Preliminary data collection** Method: Questionnaire		*n* = 300 (C)				
**Visit observation** Method: Passive observation		*n* = 60 (C)				
**Training for longitudinal data collection and analyze** Method: Face to face			*n* = 150 (C) *n* = 16 (P)			
**Longitudinal data (behavioral and developmental parameter) collection** Method: mCARE-APP/mCARE-SMS				*n* = 150 (C)		
**Group session with professionals** Method: Face to face (quarterly)				*n* = 16 (P)		
**Usability analysis** Method: Nielsen’s heuristics, System Usability Scale					*n* = 20 (C) *n* = 10 (P)	
**Visit observation** Method: Questionnaire					*n* = 120 (C)	
**Caregiver sessions** Method: Phone interview					*n* = 300 (C)	
**Data analysis**						

**Table 2.** Patient Distribution among Four Centers.

Serial	Center Name	Patients Distribution	
		Test Group	Control Group
1	The National Institute of Mental Health (NIMH)	50	50
2	The Institute of Pediatric Neuro-disorder & Autism (IPNA)	50	50
3	Autism Welfare Foundation (AWF)	25	25
4	Nishpap Autism Foundation	25	25
Total		150	150

**Table 3.** Demographic overview for test group and control group ASD children.

Features		mCARE (%)	mCARE: APP		mCARE: SMS	
			Test (%)	Control (%)	Test (%)	Control (%)
**Demographics of children**						
**Age**	2–6	25.9	7.5	6.6	4.6	7.2
	6–9	74.1	24.9	23.0	13.1	13.1
**Sex**	Male	79.7	27.2	21.6	14.4	16.4
	Female	20.3	5.2	7.9	3.3	3.9
**Education**	Never went to school	28.9	6.6	8.2	5.6	8.5
	Went to usual academic school but failed to continue study	13.1	3.6	3.0	3.6	3.0
	Went to specialized school but failed to continue study	3.0	1.0	0.3	0.3	1.3
	Currently he/she is going to usual academic school	6.2	2.3	1.6	1.6	0.7
	Currently he/she is going to specialized academic school	48.9	19.0	16.4	6.6	6.9

**Table 4.** Confidence Interval (95%) table both for the test group and control group in the Milestone Parameter improvement.

Milestone Parameter	Test Group (*N* = 150)			Control Group (*N* = 150)		
	Average Improvement Range	Lower Bound of CI	Upper Bound of CI	Average Improvement Range	Lower Bound of CI	Upper Bound of CI
**Communication**	18.3	15.4	21.1	10.8	8.5	13.0
**Daily Living skill**	28.3	26.1	30.4	25.3	22.9	27.6
**Socialization**	21.3	19.0	23.5	12.3	9.7	14.8
**Motor Skill**	23.8	21.2	26.3	17.8	15.3	20.2

**Table 5.** Confidence Interval (95%) table demographic information of the ASD children who have improved (test group, *N* = 150) in their milestone parameter.

**Factors**	**Average Range**	**Lower Bound of CI**	**Upper Bound of CI**	**Average Range**	**Lower Bound of CI**	**Upper Bound of CI**
	**Father**			**Mother**		
**Education Level of Parents (Post Graduate)**	9.8	9.1	10.4	8.3	7.4	9.1
	**Father in Service**			**Mother in Housewife**		
**Parents Occupation**	15.3	14.4	16.1	25.8	24.2	27.3
	**Specialized or Academic school going ASD children**					
**Academic information of ASD children**	19.0	18.2	19.8			
	**Urban Area**			**Nuclear Family**		
**Living Place and Family type**	25.8	24.6	26.9	25.3	24.0	26.5

**Table 6.** Timeline and Milestones of mCARE.

mCARE Timeline	Year 01				Year 02			
Timeline and milestone	Q1	Q2	Q3	Q4	Q1	Q2	Q3	Q4
Planning and advisory committee meetings								
Study Protocol Development and IRB Submission								
Aim 1: Focus group session and content development								
Aim 1: Design and development of mCARE-APP								
Aim 1: Design and development of mCARE-SMS								
Aim 1: Usability Analysis								
Aim 2: Aim 1: Design and development of mCARE-DMP								
Aim 2: Usability Analysis								
Subject Recruitment and preliminary data collection								
Longitudinal data collection, feedback and support								
Aim 3: mCARE performance and impact evaluation								
Dissemination Manuscripts								
Capacity building in mHealth research								
R01 development and submission								

## ABBREVIATIONS

NIMH: National Institute of Mental Health
IPNA: Institute of Pediatric Neuro-disorder & Autism
AWF: Autism Welfare Foundation
ISAA: Indian Scale of Autism Assessment
LMICs: Low- and Middle-Income Countries
